# Emotional Regulation and Depression: A Potential Mediator between Heart and Mind

**DOI:** 10.1155/2014/324374

**Published:** 2014-06-22

**Authors:** Angelo Compare, Cristina Zarbo, Edo Shonin, William Van Gordon, Chiara Marconi

**Affiliations:** ^1^Department of Human and Social Sciences, University of Bergamo, Piazza S. Agostino 2, 24124 Bergamo, Italy; ^2^Human Factors and Technologies in Healthcare Centre, University of Bergamo, Italy; ^3^Psychology Division, Nottingham Trent University, UK

## Abstract

A narrative review of the major evidence concerning the relationship between emotional regulation and depression was conducted. The literature demonstrates a mediating role of emotional regulation in the development of depression and physical illness. Literature suggests in fact that the employment of adaptive emotional regulation strategies (e.g., reappraisal) causes a reduction of stress-elicited emotions leading to physical disorders. Conversely, dysfunctional emotional regulation strategies and, in particular, rumination and emotion suppression appear to be influential in the pathogenesis of depression and physiological disease. More specifically, the evidence suggests that depression and rumination affect both cognitive (e.g., impaired ability to process negative information) and neurobiological mechanisms (e.g., hypothalamic pituitary adrenal axis overactivation and higher rates of cortisol production). Understanding the factors that govern the variety of health outcomes that different people experience following exposure to stress has important implications for the development of effective emotion-regulation interventional approaches (e.g., mindfulness-based therapy, emotion-focused therapy, and emotion regulation therapy).

## 1. Introduction

**Figure 1:**
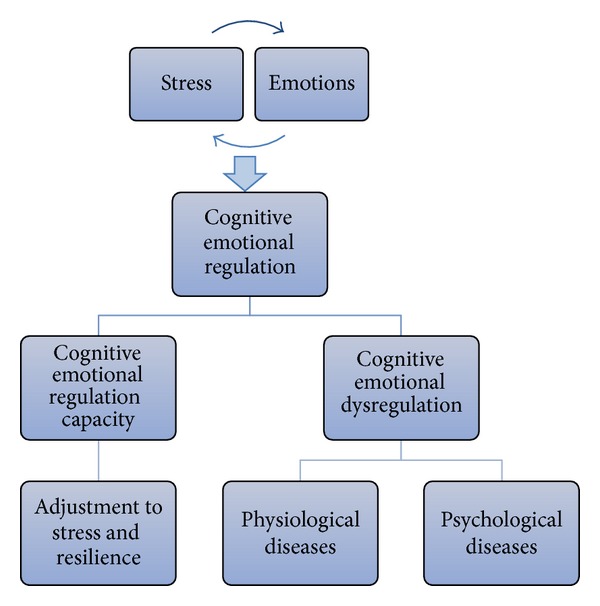
The mediating role of emotional regulation in the relationship between stress and emotions.

Exposure to stress has generally been associated with a wide range of negative outcomes, including decreased well-being and increased incidence of psychological disorders such as posttraumatic stress disorder, generalized anxiety disorder, and major depression [1, 2]. However, some individuals do not develop psychological disorders even when exposed to high levels of stress. It appears, therefore, that when faced with the same stressor, certain individuals demonstrate impaired functionality, while others show remarkable resilience. Stressful events typically elicit significant emotional responses [3, 4]. Accordingly, emotional regulation capacity has been proposed as a mediator of stress adjustment [5, 6]. According to this model, exposure to stress leads to the dysregulation of emotions, which in turn elicits negative psychological and physiological health outcomes (e.g., depression and stress-induced cardiomyopathy) (see Figure 1).

Whilst emotional regulation capacity has been proposed as a mediator in the link between emotion and psychosomatic health, a review of the empirical and theoretical literature that specifically reports on the mediating role of emotional regulation in the development of depression and subsequent additional somatic illness has not been undertaken to date. Thus, the purpose of this paper is to conduct a narrative review about the major evidence concerning the mediating role of the emotional regulation in the link between depression and physical illness. Key theories, methods of assessment, clinical and neurobiological correlates, psychological and physiological mechanisms of action, and treatment approaches are discussed.

## 2. The Emotional Regulation

**Figure 2:**
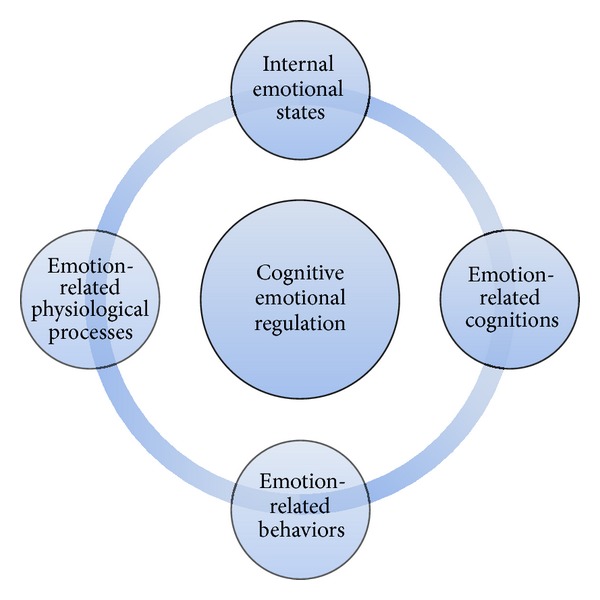
Functioning aspects involved in cognitive emotion regulation.

Emotional regulation, also known as “emotional self-regulation,” has been defined by Gross [7] as the set of cognitive processes that influence the type of emotional response, as well as how individuals experience and express these emotions. Emotional regulation is a complex process that includes the initiation, the inhibition, or modulation of the following aspects of emotion functioning [8] (see Figure 2):

1. internal emotional states (e.g., the subjective experience of emotion);
2. emotion-related cognitions (e.g., reaction to thoughts about a situation);
3. emotion-related physiological processes (e.g., autonomic arousal, hormonal reactions, etc.);
4. emotion-related behaviors (e.g., facial expressions, verbal responses, etc.).

The manner in which individuals are able to manage emotional experience to conform adaptively to a given context appears to be important to mental health [9]. Emotion regulation strategies, such as rumination, can also become maladaptive and significantly impair psychosocial functioning. Therefore, understanding when and why the regulation of emotions becomes harmful is of fundamental importance [10]. This relates to the situational and individual factors that influence cognitive emotion regulation capacity including trait and state levels of psychological, social, and somatic health [11, 12].

A model of emotional regulation process, highlighting the crucial role of emotion regulation strategies in determining the relative health-benefit of different responses to emotions, has been proposed by Gross et al. [13]. The process model of emotion regulation has been based upon the “modal model” of emotion, suggesting that a person-situation transaction that compels attention has particular meaning to an individual and gives rise to a coordinated yet flexible multisystem response to the ongoing person-situation transaction [14]. According to the modal model of emotion, emotions arise in a particular sequence that includes four key steps: (i) a situation (real or imagined) emotionally relevant; (ii) attention directed towards the emotional situation; (iii) appraisal of situation, which means that situation should be evaluated and interpreted; and (iv) emotional response to situation with changes in behavioral, experiential, and physiological response systems. The modal model of emotion suggests moreover the existence of a feedback recursively and dynamic loop from the emotional response to the situation [14].

**Figure 3:**
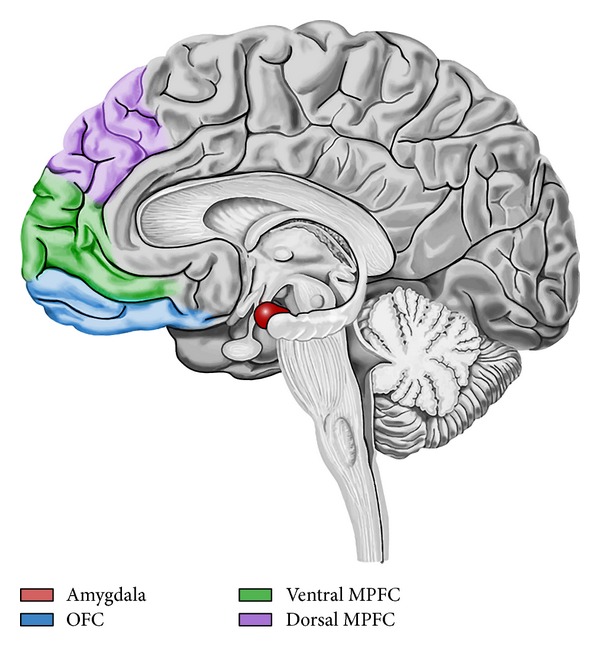
Amygdala-frontal circuit of emotion regulation. Prefrontal cortex seems to be involved in the modulation of the amygdala reactivity, a critical structure for the generation of negative emotions. During reappraisal, enhanced activation in medial prefrontal cortex (MPFC) is associated with attenuation of amygdala activation and with a reduction of negative affect intensity. Orbitofrontal cortex (OFC) coordinates the interactions between these areas.

According to the model of emotional regulation process, there are two overarching control strategies that modulate emotional experience (see Figure 3).

1. *Antecedent-Focused Regulation*. This regulation strategy occurs at an early stage in the modulation of emotional response [15] and prior to the activation of emotional and behavioral response systems [16]. Antecedent-focused regulation comprises the following types of emotion regulation strategies: (i) selecting the situation (e.g., approaching or avoiding people or situations according to their anticipated emotional impact), (ii) changing the situation (e.g., transforming the environment to alter the emotional impact), (iii) engaging attentional strategies (e.g., focusing attention towards or away from situational circumstances depending upon their emotional potency), and (iv) cognitive change (e.g., reinterpreting the situation to alter its emotional significance).
2. *Response-Focused Regulation*. This strategy is engaged at a later stage and is focused on modification of emotional output [10]. In other words, response-focused regulation refers to modifications of the physiological and observable signs of emotions after such emotions have already become manifest [15]. Response-focused regulation comprises the following types of regulatory response: (i) suppression of emotion, (ii) inhibition of emotion, (iii) emotion masking, and (iv) emotion intensification [16].

Antecedent-focused regulation, such as reappraisal, employs cognitive reevaluation strategies in order to modify situations or reformulate their emotional significance [15]. In particular, reappraisal is a cognitively oriented strategy that allows us to redefine emotional stimuli in unemotional terms or to think about a potential emotion situation in a different way to alter the emotion's impact.

Conversely, response-focused regulation, such as suppression, modulates emotional behavior via the inhibition of emotion and external behavioral signals (e.g., facial expression, verbal expressions, hand gestures, etc.). Response-focused regulation is a less adaptive strategy of regulating emotions and is associated with greater sympathetic activation of the cardiovascular system [12, 17], as well as negative outcomes such as psychopathology, social dysfunction, and depression [18]. In fact, although literature has suggested that reappraisal and suppression are both successful in reducing facial expression, only reappraisal has been shown to decrease internally felt negative emotion [7, 12, 19, 20]. Individuals who typically regulate their emotions through use of suppression report in fact less positive affect, more negative affect, less social support, and more depression [21].

## 3. Assessment of Emotional Regulation

Measures of emotional regulation typically assess the degree to which people are better able to manage their emotions [22]. The most psychometrically established and commonly employed scales for evaluating emotional regulation are as follows.

1. *The Mayer-Salovey-Caruso Emotional Intelligence Test [23]*. This scale asks respondents to evaluate the effectiveness of a range of strategies to manage emotions in different scenarios. Responses are compared with those provided by expert researchers in the field.
2. *The Emotional Regulation Questionnaire [24]*. The emotional regulation questionnaire measures the habitual use of expressive suppression and cognitive revaluation. The scale includes items related to the regulation of both positive and negative emotions [24].
3. *The Emotion Expressivity Scale [25]*. This 17-item scale measures the expression and inhibition of emotions. Emphasis is placed on the extent to which respondents suppress their emotions during social interaction [26].
4. *Difficulties with Emotion Regulation Scale (DERS) [27]*. The DERS is a brief, 36-item self-report questionnaire designed to assess multiple aspects of emotional dysregulation. Higher scores suggest greater problems with emotion regulation.
5. *Emotional Reactivity Scale (ERS) [28]*. Emotion reactivity scale (ERS) is a 21-item self-report measure of emotion sensitivity, intensity, and persistence.

## 4. Emotional Regulation and Depression

The strategies people use to regulate their emotions, and in particular negative emotions, appear to be strongly linked to several psychopathologies, such as depression. Depression is a highly prevalent emotion dysregulation disorder that impairs social skills, quality of life, and capacity to label and identify affective states. Referring to the DSM-V [29], the main criteria for a diagnosis of major depressive disorder (MDD) are

- the presence of depressed mood or a loss of interest or pleasure in daily activities for more than two weeks;
- depressed mood that represents a change from the person's baseline;
- impairment in social, occupational, and educational functions;
- the presence of specific symptoms nearly every day concerning depressed or irritable mood, decreased interest or pleasure in most activities, significant weight change or change in appetite, change in sleep, change in activity, fatigue or loss of energy, feelings of worthlessness or excessive or inappropriate guilt, diminished concentration, and suicidality.

In the last decade, evidence implicating specific emotion regulation strategies as risk factors for depression has emerged. Findings from clinical and neuroimaging studies investigating the association between emotional regulation and depression are outlined below (see Table 1).

## 5. Clinical Studies

A number of clinical studies have shown that inappropriate or ineffective emotion regulation is a critical component in the development and maintenance of depression and anxiety disorders [30–35]. Depression is also associated with impaired cognitive control such as difficulty in accepting and processing negative material [36]. In turn, this reduced ability to process negative material is associated with increased rumination, impaired cognitive reappraisal, and increased expressive suppression [36]. Clinical studies have also demonstrated that depressed patients are negatively biased in their recognition of emotion, which increases significantly according to diagnostic severity [37].

## 6. Neurobiological and Neuroimaging Studies of Circuit of Emotion Regulation

Several neurobiological studies have focused on the link between emotional regulation and depression. Evidence implicates dysfunctions in the brain serotonergic system in the pathophysiological mechanism of major depressive disorder (MDD). Knockout mice with targeted deletion of genes involved in the mediation of serotonergic transmission (i.e., removal of tryptophan hydroxylase 2 (Tph2 KO) which is a rate-limiting enzyme for serotonin (5-HT) synthesis) have demonstrated the important role played by the brain serotonergic system in emotion dysregulation [38]. Endogenous opioid neurotransmission that activates mu-opioid receptors has also been found to be involved in emotion regulatory processes and is likewise implicated in the etiology of MDD [39]. More specifically, negative mood is associated with statistically significant decreases in mu-opioid receptor binding potential in the left inferior temporal cortex of patients with MDD [39].

Neuroimaging studies have positively correlated MDD and ineffective emotion regulation with heightened amygdala response [40]. Neuroimaging studies also demonstrate that the prefrontal cortex (PFC) is involved in mediating important higher-order cognitive processes and that the level of activation of the ventral prefrontal cortex reflects the degree of emotional modulation and cognitive control on cognitively demanding goal-directed tasks [41]. Dysfunction in this area of the brain has been hypothesized to explain the pathological inability of the brain to regulate and suppress the heightened affective responses to negative affect-eliciting stimuli associated with depression [41]. In fact, PFC activation is strongly influenced by emotional reactions through its functional interaction with the amygdala and the striatal circuitry (areas of the brain involved in emotion and reward processing) [42].

In Figure 3, we describe the amygdala-frontal circuit of emotion regulation. While the amygdala is a critical region to the generation, expression, and experience of negative emotions, some frontal cortical regions are thought to be involved in the modulation of amygdala reactivity and the mediation of effective emotion regulation, with their top-down inhibitory effect on the amygdala. Moreover, stronger coupling exists between amygdala and these areas of the prefrontal cortex (PFC) during conscious emotion regulation, like reappraisal: attenuation of amygdala activation is associated with enhanced activation in ventromedial PFC and dorsal medial PFC [43]. The medial PFC (MPFC) has been linked to the integration between emotion and cognition and to the processes that underlie cognitive reappraisal. A functional distinction has been posited between ventral and dorsal MPFC: ventral MPFC seems to be involved in appraisal for emotional salience of stimuli, while dorsal MPFC seems to be involved in regulation of appropriate behaviors following appraisal. Orbitofrontal cortex (OFC) works as an important relay or coordination region for amygdala and MPFC interactions. Greater functional connectivity between amygdala and OFC/dorsal MPFC during reappraisal is associated with less intensity of negative affect.

## 7. Rumination: A Maladaptive Emotional Regulation Strategy

Depressed individuals experience a range of maladaptive cognitions that may lead to underregulation. Rumination, the most common maladaptive emotional regulation strategy in depressed individuals, can be defined as an emotional process characterized by repetitive, unwanted, past-oriented negatively inclined thoughts [44, 45]. Rumination is generally considered to be maladaptive and has been implicated in the exacerbation and maintenance of a variety of adverse mental health outcomes, including depression [46, 47]. Rumination is a process related to worry and refers to a cognitive focus on real or imagined upcoming negative events [48]. Worry is a form of fear process and is associated with anxiety, apprehension, and general tension [49]. Rumination and worry are similar in that they are both characterized by repetitive negative thought [45]. For individuals who ruminate or who mentally rehearse past stressful events, the physiological effects of stressors are often more enduring [37] (e.g., continued reflection on a bygone argument may cause stress hormones to continue to circulate in the body long after an argument has ended). The evidence implicates impaired cognitive control as a crucial process underlying ruminative thinking and suggests that it may be an important vulnerability factor for depression [50].

The determinants of rumination are multifaceted. Individual and situational differences may play a role in triggering and maintaining ruminative thought [51], and certain individuals are more prone to perseveration than others [44]. The evidence indicates that repeated psychosocial stress in early life has a significant impact on behavior and neural functioning—both of which are risk factors for depression [52]. Furthermore, it has been hypothesized that early-life adversity may result in a reduced capacity for cognitive control in response to a repeated stressor, particularly in individuals who develop maladaptive emotional processing strategies (i.e., trait rumination). In contrast, individuals who were exposed to early-life adversity but developed adaptive emotion processing skills (e.g., high levels of trait mindfulness) typically exhibit enhanced capacity for cognitive control during adulthood [52].

## 8. Physiological Mechanisms of Rumination

Rumination has been shown to induce prolonged activation of the hypothalamic pituitary adrenal (HPA) axis, and, for those who ruminate excessively or repeatedly, sustained elevations in cortisol may incur deleterious health consequences [51]. Examples are acute changes in the cardiovascular system and acceleration of atherosclerotic process [53]. The HPA axis is a slow-acting stress response system, whose activation triggers a cascade of events which begins with the secretion of corticotropin-releasing hormone by the hypothalamus and culminates in the release of glucocorticoids (i.e., cortisol) by the adrenal glands into the bloodstream [54]. The HPA axis is a major stress response system that is critical for survival and adaptation and may be particularly relevant in understanding the adverse effects that stressors have on somatic and psychological health. Subsequent and repeated recall of a stressor, which is symptomatic of both rumination and depression, could reactivate the stress response during otherwise nonstressful situations. In fact, evidence suggests that perseverative cognition, such as rumination, may prolong cardiovascular-related physiological activation [55]. Although release of cortisol by the HPA in response to certain stressors may be adaptive in the short term [56], prolonged exposure to cortisol from exaggerated, extended, or repeated activation of the HPA axis is likely to be maladaptive. Indeed, a range of disorders, including cardiovascular disease, have been associated with persistent HPA axis activation [57]. Furthermore, HPA overactivation and elevated basal cortisol levels have been linked to avoidance, withdrawal, and negative emotions such as anxiety and depression [58–63].

Recent studies investigating how the neural correlates of personality regulate emotion processing in the brain have associated the inferior frontal gyrus (IFG) (Brodmann area 45) with trait high levels of rumination [58]. The IFG asserts an important regulatory role in top-down control processes that lead to increases in subcortical activation [64]. Therefore, it is reasonable to assume that, by promoting ruminative thinking, emotional suppression is likely to increase positive connectivity between subcortical limbic activation and the IFG.

## 9. Emotional Regulation Role in the Link between Depression and Illness

Literature has suggested that depression, through neurobiological and behavioral processes, may affect physical health and induce physical illness. In particular, depression has been found to be the primary risk factor for cardiac diseases [65]. Depression has been in fact linked to (i) several biological mechanisms, such as inflammatory and immune processes, alterations in activating HPA, variability in heart rate, increased activity of the sympathoadrenal and pituitary-adrenal axes, reduction in circulating endothelial progenitor cells, increase of cortisol and catecholamine levels, alteration of activities of autonomic nervous system, and oxidation processes [66–69] and (ii) several unhealthy lifestyles, such as increased consumption of tobacco, alcohol, and illicit substances, reduced physical activity, overeating, and no medical adherence [68].

Due to the well-known and yet discussed existence of dysfunctional emotional regulation in depression and rumination, a mediating role of emotional regulation in the link between depression and somatic disease may be suggested. Emotional regulation may indirectly affect both the etiology and the prognosis of a physical illness. According to inhibition theory, keeping emotions inside will lead to long-term health problems because it requires continuous physiological work [70]. Several studies have in fact suggested that stronger mood regulation expectancies are associated with lower dysphoria and somatic symptoms in college students and caregivers of chronic diseases [71, 72]. Several studies have shown that different styles of emotion regulation have different relationships with health [12, 73, 74]. In particular, alexithymia, emotional control, and ambivalence have been significantly related to more psychological, social, and physical distress in both healthy and chronically ill populations [75–77]. In contrast, emotional orientation and expression have shown beneficial effects and positive health consequences [78–81].

## 10. Psychological Treatments Focusing on Emotional Regulation

Current neuroscientific evidence confirms that both emotion and cognition are interacting yet distinct functions which communicate via bidirectional neural connections between the neocortex and emotional centers [82]. These connections allow emotion-related input to modulate cortical activity and cognitive input from the cortex to modulate emotional processing. Understanding how cognitive and emotional systems interact has significant implications for emotion regulation interventions. For example, it has been suggested that intervening at the level of the emotional system itself is a more direct, efficient, and powerful way to override and transform the maladaptive patterns that underlie unhealthy psychological, behavioral, and physiological stress responses [82].

The activation of positive emotions can play a critical role in breaking the stress cycle by effectively transforming stress at its source [83]. Positive emotions have been linked to improved health and increased longevity. They have also been shown to beget increases in cognitive flexibility, creativity, receptivity, innovative problem solving, and psychological resilience in the face of adversity. Furthermore, positive emotions influence interpersonal behavior and promote helpfulness, generosity, and cooperation [61–63]. In other words, positive emotions play a critical role in effective adaptation to life's challenges [84–86]. The literature also suggests that emotional regulation strategies such as positive reappraisal and curtailed rumination exert a protective influence over relapse or exacerbated symptoms in patients with depression and anxiety disorders [87]. Above all, the most common therapies focusing on emotional regulation are presented.

## 11. The Emotion-Focused Therapy

To be able to effectively regulate emotions, individuals need to be assessed and trained in evidence-based emotion management strategies. In a treatment protocol developed by Greenberg and known as emotion-focused therapy [88], individuals are taught to enhance their awareness of emotion regulation strategies and of emotional processes more generally. Emotion-focused therapy is based on an awareness-enhancing strategy operationalized in an intervention known as process experiential therapy [89], which aims to help people develop emotional intelligence and effective emotion regulation processes. Greenberg [88] proposes three key principles that underlie emotional change.

1. *Emotion Awareness*. This relates to a greater awareness of emotional experiences and the information they provide, as well as a greater understanding of the behaviors that result from such emotions. Increased emotional awareness promotes the creation of new meanings and helps people to develop new narratives to explain their experiences.
2. *Emotion Regulation*. In order to reach a state of connection with their own emotional experience, it is necessary to increase people's capacity for self-calm (e.g., by regulating the heartbeat and breathing). Reduced autonomic and psychological arousal allows people to derive insight and knowledge from their emotional processes and to learn how to handle emergency signals and situations.
3. *Changing Emotion with Emotion*. A nonadaptive affective state can be transformed into an adaptive one. This change is made possible by relaxation exercises or through the deliberate induction of positive emotions [16].

The main objective of emotion-focused therapy is to help individuals modify internal affective states, the core beliefs that underlie these states, and the behaviors that they elicit. Emphasis is placed on intervening before the emotion-eliciting event takes place. This occurs via an increased ability to analyze and be aware of emotions which improves a person's ability to handle internal and external stressors. Emotion-focused therapy has been widely used in interventions aimed at the treatment of depression [16].

## 12. The Mindfulness-Based Cognitive Therapy

A further innovative interventional approach that has recently been deployed in clinical contexts is that of mindfulness training. Mindfulness derives from Buddhist meditation practice and is fundamentally concerned with developing and open and unbroken awareness of present moment cognitive-effective and sensory experience. According to Compare et al. [67], mindfulness effectuates a greater perceptual distance from distorted cognitive and affective processes and this meta-awareness facilitates the regulation (i.e., via the nonreactive observance) of habitual maladaptive responses. Via the regulation of attention, mindfulness empowers participants to cultivate a new relationship with internal and external experiences and in a way that does not encourage avoidance, overengagement, or elaboration [90].

During the last two decades, a credible evidence base has emerged supporting the utilization of mindfulness meditation as an emotion regulation strategy. Indeed, a mindfulness approach known as mindfulness-based cognitive therapy (MBCT) is now advocated by the National Institute for Health and Clinical Excellence (NICE) and the American Psychiatric Association for the treatment of specific forms of depression. Evidence has also shown that increases in levels of dispositional mindfulness are significantly correlated with reductions in avoidance and rumination [91].

Increased breath awareness (a fundamental component of mindfulness meditation) has been shown to reduce autonomic and psychological arousal, and this increased capacity to remain calm can help individuals to respond more adaptively to internal and external stressors [70]. Negative thinking and ruminative thinking are often employed as maladaptive means of escaping from negative affective states such as guilt, depression, and anxiety [71]. The increased levels of self-awareness cultivated during mindfulness practice can confer a greater capacity to label and therefore modulate these affective states [71]. In other words, rather than avoiding distressing feelings and thoughts by engaging in ruminative thinking and/or maladaptive behaviors, mindfulness encourages people to objectify any dysfunctional cognitive and affective processes by seeing them as passing phenomena.

Another important mechanism by which mindfulness is believed to modulate negative affective states is via the cultivation of self-compassion and compassion. Research has shown that mindfulness leads to a greater awareness of the individuals own suffering and psychological distress, and this helps to instil a greater appreciation of the suffering of others [70, 72]. Accordingly, greater levels of self-compassion and compassion are thought to lead to improvements in pain and emotion tolerance due to individuals being able to assign greater levels of perspective to their emotional and/or somatic pain (i.e., by appreciating that they are not the only person experiencing psychological/somatic distress) [72].

## 13. The Emotion Regulation Therapy (ERT)

The emotion regulation therapy (ERT) is a recent treatment for generalized anxiety disorder (GAD) that integrates components of cognitive-behavioral, acceptance, dialectical, mindfulness-based, experiential, and emotion-focused treatments [92, 93]. The ERT is based on the emotion dysregulation model of GAD that suggests that GAD subjects have difficulties in the modulation of emotions, showing difficulty in identifying, describing, and clarifying the motivational content of emotion. GAD subjects utilize in fact worry as a maladaptive cognitive control strategy in order to reduce and control an aversive or uncertain emotional state. ERT aims, then, to enhance knowledge, acceptance, utilization, and management of emotions addressing cognitive, emotional, and contextual factors that may contribute to maladaptive responses.

ERT program includes 20 sessions of 50–60 minutes, divided in 16 weeks and four phases. The first phase focuses on psychoeducation about GAD, functional patterns of worry and emotions in various situations, and self-monitoring of worry episodes. The second phase focuses on the development of somatic awareness and emotion regulation skills. The third phase focuses on the application of skills during exposure to emotionally evocative themes. The fourth and final phase focuses on terminating the therapeutic relationship, relapse prevention, and future goals [35].

The main aim of ERT for GAD subjects is to learn the ability to effectively increase or decrease attendance to emotional experience, tolerate distress, and adapt to social environment. In specific, ERT aims to teach GAD subjects to: (i) identify, differentiate, and describe their emotions; (ii) increase both acceptance of affective experience and ability to adaptively manage emotions; (iii) decrease use of emotional avoidance strategies, such as worry; (iv) increase ability to utilize emotional information in identifying needs, making decisions, guiding thinking, motivating behavior, and managing interpersonal relationships and other contextual demands [35].

## 14. Conclusions

Based on an extensive examination of the empirical and theoretical literature related to emotional regulation, it appears that a strong association exists between emotional regulation and depression and that emotional regulation mediates the role between depression and further psychological and/or somatic illness. It is concluded that when adaptive emotional regulation strategies (e.g., reappraisal) are employed, there is reduced likelihood of stress-elicited emotions leading to mental or physical disorders.

Although there is some evidence that suggests the involvement of frontal cortical regions in the modulation of amygdala reactivity towards emotion regulation, interregional connectivity between amygdala and prefrontal cortex in the context of affect regulation remains unknown. Further studies are needed to better explain the neural circuit connecting amygdala and PFC in emotion regulation.

Conversely, emotional dysregulation (e.g., rumination, suppression, etc.) appears to be influential in the pathogenesis of psychological disease, such as depression. More specifically, the evidence suggests that depression and rumination affect both cognitive (e.g., difficulty in processing negative information) and neurobiological mechanisms (e.g., HPA axis overactivation and higher rates of cortisol production).

Given the height link between depression, emotional regulation, and somatic diseases, future research and clinical studies should focus on the specific role of emotional regulation in affecting both depression and somatic diseases in order to highlight the specific mental and biological processes that lead to illness. Understanding the factors that govern the variety of health outcomes that different people experience following exposure to stress will have important implications for the identification and ongoing validation of effective emotion-regulation interventional approaches (e.g., mindfulness-based therapy, emotion-focused therapy, emotion regulation therapy, etc.).

## Figures and Tables

**Table 1:** Emotional regulation and depression link in clinical and neuroimaging studies.

Emotional regulation and depression	
Clinical studies	Neuroimaging studies
Depressed patients show (i) inappropriate or ineffective emotion regulation; (ii) difficulties in cognitive control; (iii) difficulties in processing negative material (which leads to greater rumination, less use of reappraisal strategies, and more use of expressive suppression); (iv) negative self-report biases.	Depressed patients show (i) dysfunctional stress responses; (ii) decrease in mu-opioid receptor binding potential in the left inferior temporal cortex; (iii) abnormal amygdala activation; (iv) dysfunctional ventral prefrontal cortex activation.
